# *Klebsiella pneumoniae* Induces Inflammatory Bowel Disease Through Caspase-11–Mediated IL18 in the Gut Epithelial Cells

**DOI:** 10.1016/j.jcmgh.2022.11.005

**Published:** 2022-11-25

**Authors:** Qianjin Zhang, Xiaomin Su, Chunze Zhang, Wei Chen, Ya Wang, Xiaorong Yang, Dan Liu, Yuan Zhang, Rongcun Yang

**Affiliations:** 1 Translational Medicine Institute, Affiliated Tianjin Union Medical Center of Nankai University, Nankai University, Tianjin, China; 2 State Key Laboratory of Medicinal Chemical Biology, Nankai University, Tianjin, China; 3 Department of Immunology, Nankai University School of Medicine, Nankai University, Tianjin, China; 4 Affiliated Tianjin Union Medical Center of Nankai University, Nankai University, Tianjin, China

**Keywords:** *Klebsiella pneumoniae*, Inflammatory Bowel Diseases, Caspase-11, APC, Allophycocyanin, CARD, Caspase Recruitment  Domain, CFU, colony-forming unit, DAI, Disease Activity Index, DAPI, 4′,6-diamidino-2-phenylindole, DMEM, Dulbecco’s modified Eagle medium, DSS, dextran sodium sulfate, ELISA, enzyme-linked immunosorbent assay, FBS, fetal bovine serum, IBD, inflammatory bowel disease, IFNγ, interferon γ, IL, interleukin, *KLP*, *Klebsiella pneumoniae*, *KLPJ*, *Klebsiella pneumoniae J*, KO, knockout, LPS, lipopolysaccharide, NK, natural killer, NLR, nucleotide-binding domain leucine-rich repeat containing, NLRC4, NLR family CARD domain containing 4, PBS, phosphate-buffered saline, PE, Phycoerythrin, SPF, specific-pathogen free, Th1, T-helper 1, UC, ulcerative colitis, WT, wild-type

## Abstract

**Background & Aims:**

*Klebsiella pneumoniae* (*KLP*), a Gram-negative bacterium belonging to the family *of Enterobacteriaceae*, is a common cause of antimicrobial-resistant opportunistic infections in hospitalized patients. *KLP* can colonize in the human gastrointestinal tract, especially in patients with inflammatory bowel diseases. However, effects of *KLP* on the onset and development of inflammatory bowel disease remain unclear.

**Methods:**

We analyzed the relationship between Mayo indexes of ulcerative colitis and *KLP* using quantitative reverse-transcription polymerase chain reaction and endoscopy. Using *caspase-1/11-/-*, *NLRP3-/-*, *NLRC4-/-*, interleukin (*IL*)*18-/-*, and *IL22-/-* mice, we showed that *KLP* could induce colitis through caspase-11–mediated release of mature IL18. Through in vitro gut organoid culture, we determined the mechanism for *KLP* to induce colitis.

**Results:**

We first found that there was a positive relationship between the Mayo indexes of ulcerative colitis and *KLP*. Then, we isolated a strain of *KLP*, named *Klebsiella pneumoniae J* (*KLPJ*), from the colon tissues of patients with colitis. This strain of bacteria could induce the production of mature IL18 in colon epithelial cells and gut organoids, and also induce colitis and promote dextran sodium sulfate–mediated colitis. Using *caspase-1/11-/-*, *NLRP3-/-*, *NLRC4-/-*, *IL18-/-*, and *IL22-/-* mice, we showed that *KLPJ*-mediated colitis occurred through activation of caspase-11, and was dependent on IL18 and partly on IL22. Our data also showed that lipopolysaccharide from *KLPJ* could bind with caspase-11 to induce mature IL18 in mouse and human colon organoids.

**Conclusions:**

*KLPJ* from the colon tissues of patients with ulcerative colitis can colonize the colon, activate caspase-11 inflammasomes, and contribute to intestinal inflammation.

## Graphical abstract

**Figure:**
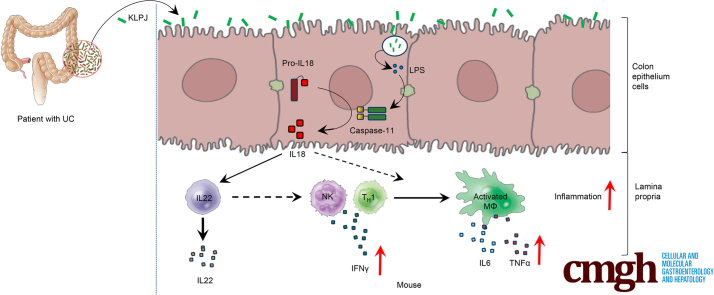

**Summary**

There is a positive relationship between Mayo indexes of ulcerative colitis and *Klebsiella pneumoniae*. *K pneumoniae* can induce colitis through activation of caspase-11 and release of interleukin 18 in gut epithelial cells.

Inflammatory bowel disease (IBD) is a chronic and relapsing disorder that can affect the gastrointestinal tract,^1^^,^^2^ including 2 main subtypes: Crohn’s disease (CD) and ulcerative colitis (UC). Chronic colonic inflammation also increases the risk of developing colorectal cancer.^3^^,^^4^ Disruption of homeostasis between the host immune system and the intestinal microbiota can lead to IBD. Gram-negative bacteria such as *Bacteroides fragilis*, *Fusobacterium nucleatum*, *Escherichia coli*, and *Helicobacter pylori* have been linked to IBD, including Crohn’s disease, UC, and colorectal cancer.

*Klebsiella pneumoniae* (*KLP*), another gram-negative bacterium that belongs to the family of *Enterobacteriaceae*, is a common cause of antimicrobial-resistant opportunistic infections in hospitalized patients.^5^ The most common manifestations are pneumonia and urinary tract and wound infections.^5^^,^^6^ However, *KLP* also can act as a true pathogen to cause severe community-acquired infections, including endophthalmitis, pneumonia, necrotizing fasciitis, nonhepatic abscess, meningitis, and pyogenic liver abscess in the absence of biliary tract disease.^5^^,^^6^ Studies in recent years have begun to recognize the effects of *KLP* on the causation and progression of gastrointestinal tract diseases. Multiple groups have shown that *KLP* can colonize the intestine and has detrimental effects in colitis models.^7^, ^8^, ^9^, ^10^, ^11^, ^12^ They not only shed light on host colonization by *KLP* and identify potential colonization factors such as genetic factors that contribute to high-density persistence of *KLP* in the intestine,^9^ but also the importance of xylose metabolism in *KLP* colonization in mice.^11^ *Klebsiella* microorganisms also are isolated from the stools of both healthy individuals and patients who had hemorrhagic colitis and diarrhea.^13^^,^^14^ These *KLP* also can colonize in the human gastrointestinal tract, especially in patients with IBD,^15^^,^^16^ and result in a continuous cycle of insult to the colonic mucosa by various cytokines, eventually leading to the development of Crohn's disease.^14^ However, how *KLP* affects the onset and development of colitis remains unclear.

Interleukin (IL)18 has been postulated to be a key determining factor in IBD.^17^^,^^18^ This pathogenic role of IL18 correlates with clinical observations. An increase in both epithelial and hematopoietic IL18 expression and its bioreactivity have been shown in patients with increased severity of IBD.^19^^,^^20^ IL18 initially is synthesized as an inactive precursor molecule. Its secretion in epithelial cells is controlled by inflammasomes, multiprotein complexes basically composed of the nucleotide-binding domain leucine-rich repeat containing (NLR) family and cysteine protease caspase-1,^21^ which can cleave pro-IL18 into a functional mature bioactive cytokine.^22^^,^^23^ IL18 plays an important role in the induction of interferon γ (IFNγ), increasing natural killer (NK) cell activity and T-cell proliferation.^24^ Here, we found that isolated *K pneumoniae* from the colon tissue of patients with UC could induce colitis and promote dextran sodium sulfate (DSS)-mediated colitis through caspase-11–mediated IL18.

## Results

### *K pneumoniae* Isolated From the Colon Tissues of Patients With UC Induces Secretion Of Mature IL18 in the Colon

**Figure 1:**
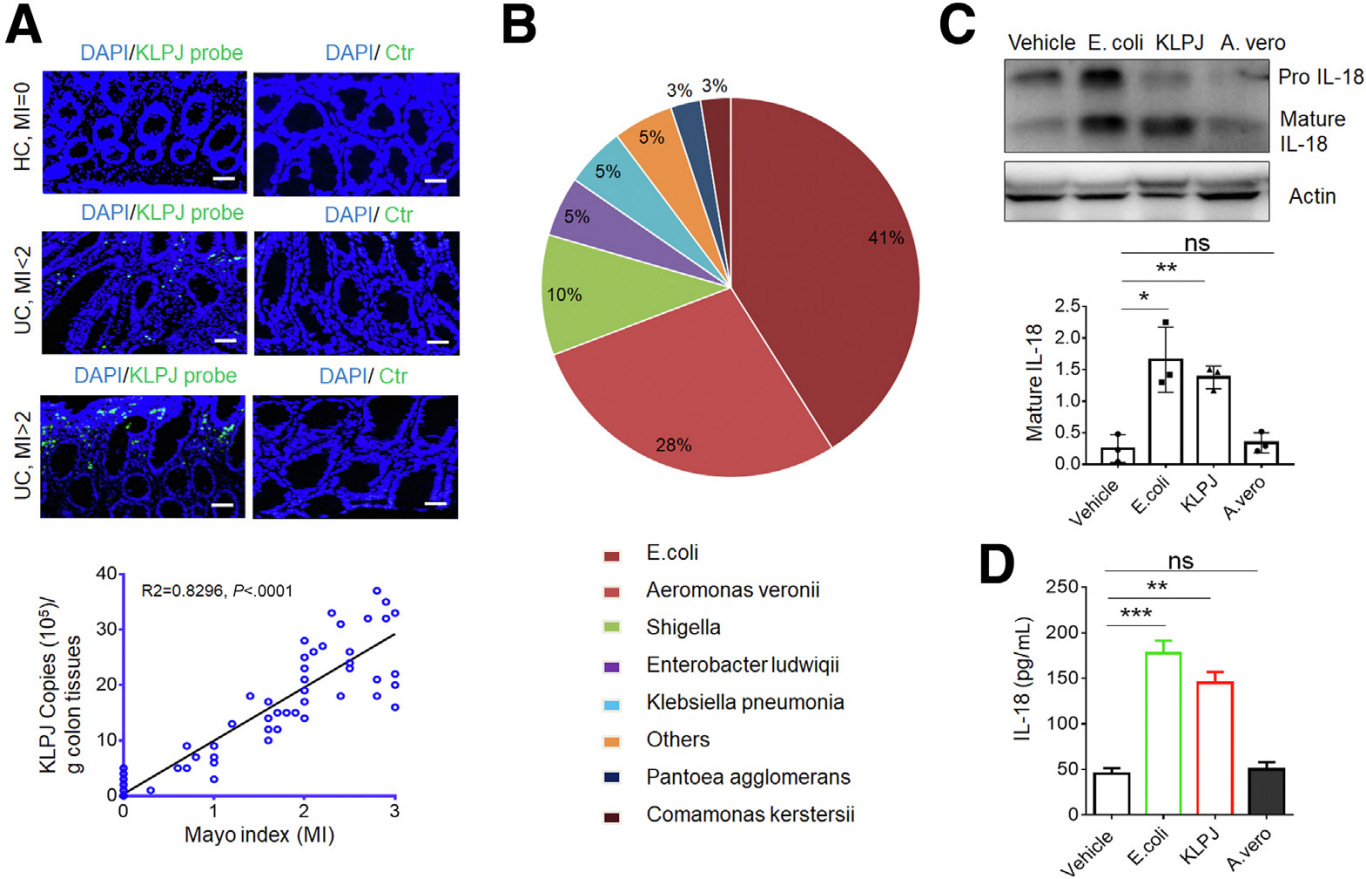
***KLPJ* isolated from the colon tissues of patients with UC induces production of mature IL18 in colon epithelial cells.** (*A*) Hybridization of fluorescent *KLPJ* probe and relationship between the Mayo index and *KLP* copies in the colitis tissues of patients with UC (n = 50). The relationship between the Mayo index and *KLP* copies was analyzed using correlation and regression (GraphPad). R2, Pearson correlation coefficient. Green, *K pneumoniae* probe; blue, nuclei. Control probes indicate fluorescence-labeled control probes. *Scale bar*: 40 μm. (*B*) Composition of bacteria in the colitis tissues of patients with UC. A total of 628 bacterium clones from the colon tissues of 12 patients with UC were cultured and analyzed. (*C*) Western blot of IL18 in the colon epithelial cells after exposure to *KLPJ*. Colons were isolated and exposed to different bacteria, and then colon epithelial cells were analyzed. (*D*) ELISA of IL18 in the colon contents after exposure to *KLPJ*. Colons were exposed to different bacteria, and then colon contents were analyzed. Student *t* test. ∗*P* < .05, ∗∗*P* < .01, and ∗∗∗*P* < .001. A. vero, *A veronii*; Ctr, control; HC, healthy colon tissues; MI, Mayo index; UC, tissues of ulcerative colitis; vehicle, control.

*KLP* colonizes in the human gastrointestinal tract, especially in patients with IBD.^15^^,^^16^ The 16S ribosomal RNA sequencing analyses of colonic contents also showed that there was a larger amount of *KLP* in patients with UC.^14^^,^^25^ We also found that a positive relationship existed between the Mayo indexes of UC^26^ and *KLP* (Figure 1*A*). Thus, we next examined whether the *K pneumoniae* in UC tissues could induce colitis. The colon tissues from 12 patients with UC were collected, and hundreds of bacterium clones were cultured. After sequencing, these clones were composed mainly of *E coli*, *Aeromonas veronii*, *Shigella*, *Enterobacter ludwiqii*, *K pneumoniae*, and others (Figure 1*B*). Because multiple strains of *KLP* potentially are related to different diseases,^5^^,^^6^ we also performed phylogenetic analysis using MEGA-X and found that this dominant strain of *KLP* belonged to *K pneumoniae JCM1662*, which was named *K pneumoniae J* (*KLPJ*). Colons from mice were infused and stimulated with the isolated *KLPJ* or *E coli* 0160:H7, which was isolated from the colon tissue of patients with UC.^27^^,^^28^ Then, colon epithelial cells were isolated for Western blot, and the colon contents were collected for mature IL18. Both *E coli* 0160:H7 and *KLPJ* could induce production of mature IL18 (Figure 1*C* and *D*). Because IL18 in gut epithelial cells is a key determining factor in IBD,^17^^,^^18^ the isolated *KLPJ* might be related to IBD.

### Isolated *K pneumoniae* Induces and Promotes Colitis

**Figure 2:**
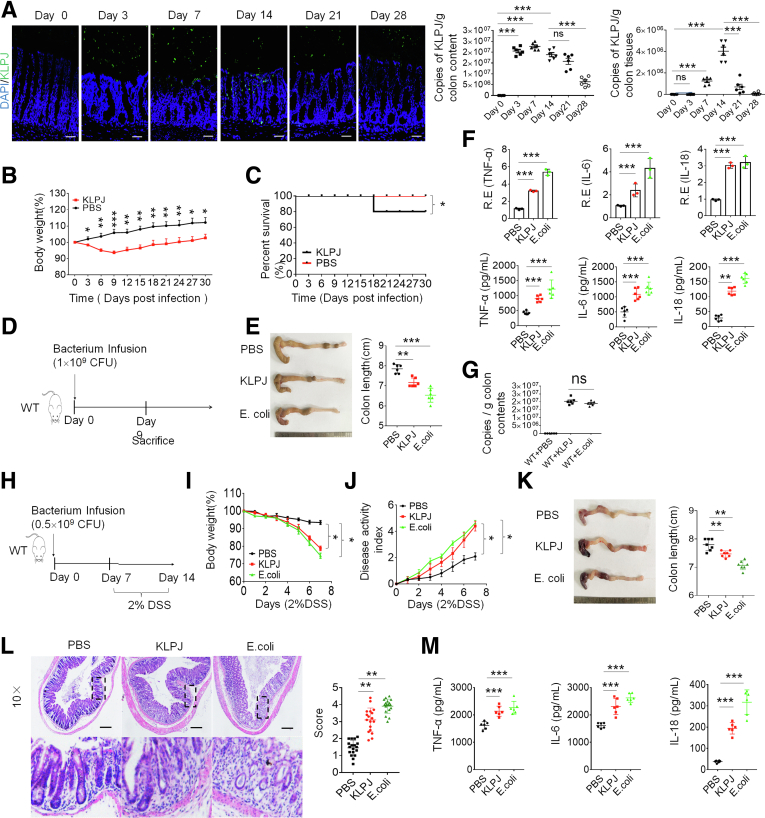
**Isolated *KLPJ* induce colitis in WT mice.** (*A*) Hybridization of fluorescent *KLPJ* probe and *KLPJ* copies in colon contents and colon tissues of WT mice (n = 6) at different time points after infusing *KLPJ*. (*B*) Body weights of mice after gavage by *KLPJ* (n = 9). (*C*) Survival rate of SPF mice after gavage by *KLPJ* (n = 12). (*D*) Schematic of the experiment for *KLPJ*-induced colitis. (*E*) Colon length in mice after gavage by *KLPJ* (n = 6). (*F*) Quantitative reverse-transcription polymerase chain reaction and ELISA of tumor necrosis factor α (TNFα), IL6, and IL18 in the mixed samples of colon tissues of mice (n = 6). (*G*) Bacterium copies in colon contents of mice on day 7. (*H*) Schematic of the experiment for *KLPJ*-induced colitis after DSS. (*I*) Body weights of mice after DSS (n = 9). (*J*) DAI of mice after DSS (n = 9). (*K*) Colon length in mice after DSS (n = 6). (*L*) H&E staining of colon tissues of mice after DSS (n = 6). (*M*) ELISA of TNFα, IL6, and IL18 in the colon tissues of mice after DSS (n = 6). Mice with (*KLPJ* or *E coli*) or without (PBS) gavage of *KLPJ* or *E coli*. PBS represents control. (*B*, *I*, and *J*) One-way analysis of variance test; (*C*) Wilcoxon test; (*A*, *E*, *F*, *G*, *K*, and *M*) Student *t* test; and (*L*) Mann–Whitney *U* test. (*A* and *L*) *Scale bar*: 40 μm. ∗*P* < .05, ∗∗*P* < .01, and ∗∗∗*P* < .001. Data are representative of at least 2 experiments. R.E., relative expression.

We next examined whether this isolated *KLPJ* could colonize in the colon of specific-pathogen free (SPF) mice. Data showed that this bacterium could not only colonize in the colon but also co-exist with other microbiota after gavage (Figure 2*A*), consistent with other reports that the *Klebsiella*/*Enterobacteria* species can translocate to the lower digestive tract and ectopically colonize therein.^15^^,^^16^^,^^29^ Notably, the number of *KLPJ* in the mice after gavage gradually began to decrease in both colon contents and in colon tissues after day 14 (Figure 2*A*). Furthermore, mouse body weights also did not continue to decrease and the death rate did not increase after day 14 (Figure 2*B* and *C*). All of these imply that a *KLPJ* infection may be self-limiting.

Because *KLPJ* can colonize in the colon, we investigated whether this strain of *KLPJ* could cause colitis. After oral gavage using isolated *KLPJ* (10^9^ colony-forming units [CFU]/mouse), a larger amount of lost body weight and shorter colon length were observed in the *KLPJ*-infused mice compared with the control group, although they were more remarkable in mice infused with *E coli* (Figure 2*D* and *E*), which were isolated from mice with colitis,^27^ implying that *KLPJ* could induce IBD. Indeed, there also was increased transcription and expression of inflammatory cytokines (Figure 2*F*). Equal amounts of bacteria in the colon of infused mice were detected (Figure 2*G*). We also examined whether *KLPJ* could promote DSS-mediated colitis (Figure 2*H*). The body weights, disease activity index (DAI), colon lengths, and H&E staining indicated that the colitis was more severe in the *KLPJ*-infused mice (Figure 2*I*–*L*). There also was more expression of inflammatory cytokines in the *KLPJ*-infused mice (Figure 2*M*).

**Figure 3:**
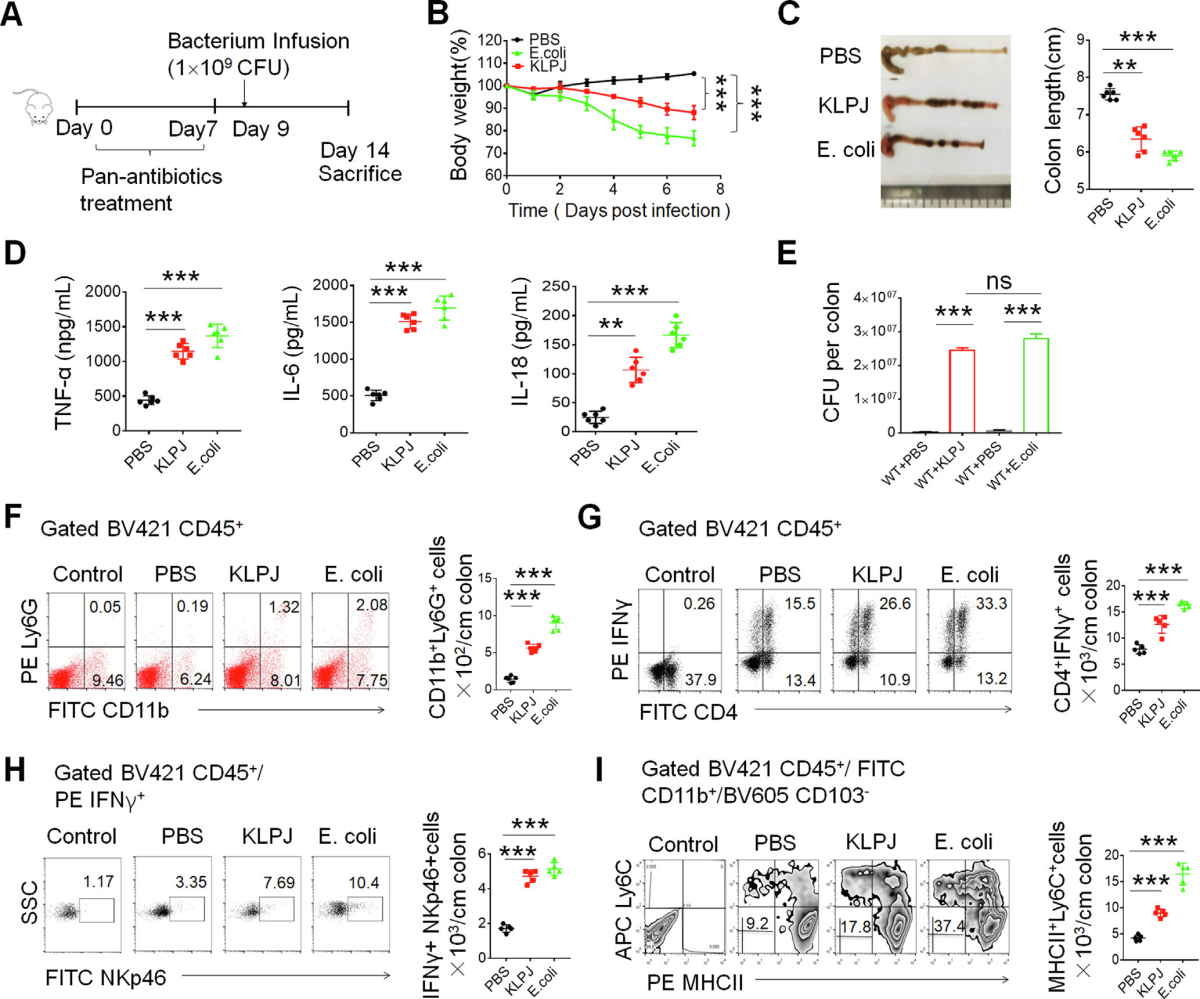
**Isolated *KLPJ* induces colitis in pan-antibiotics–treated mice.** (*A*) Schematic of the experiment for *KLPJ*-induced colitis. (*B*) Body weights of mice (n = 9). (*C*) Colon length in mice (n = 6). (*D*) ELISA of tumor necrosis factor α (TNFα), IL6, and IL18 in the colon tissues of mice (n = 6). (*E*) CFU of *KLPJ* or *E coli* in the colon contents of mice. For CFU in the bacteria-infused mice, 10^9^ bacteria were orally infused and then CFU were counted after 7 days. (*F*) Flow cytometry of CD11b^+^Ly6G^+^ cells in the colon tissues of mice (n = 5). Control indicates Ly6G isotypic control. (*G*) Flow cytometry of CD4^+^IFNγ^+^ cells in the colon tissues of mice (n = 5). Control indicates IFNγ isotypic control. (*H*) Flow cytometry of NKp46^+^IFNγ^+^ cells in the colon tissues of mice (n = 5). Control indicates isotypic control. (*I*) Flow cytometry of CD11b^+^MHCII^+^Ly6C^+^ inflammatory macrophages in the colon tissues of mice (n = 5). Control indicates isotypic control. Mice with (*KLPJ* or *E coli*) or without (PBS) gavage of *KLPJ* or *E coli*; PBS indicates control. (*B*) One-way analysis of variance test; (*C–I*), Student *t* test. ∗∗*P* < .01 and ∗∗∗*P* < .001. Data are representative of at least 2 experiments. FITC, fluorescein isothiocyanate; SSC,SideScatter.

There are 2 possibilities for *KLPJ*-mediated colitis. One is that *KLPJ* directly mediates colitis, another is that *KLPJ* indirectly causes colitis. To eliminate the indirect factors, we used a pan (ampicillin, vancomycin, neomycin, and metronidazole)-antibiotics–treated mouse model (Figure 3*A*). After pan-antibiotics–treated mice were infused using isolated *KLPJ* (10^9^ CFU/mouse), shorter colon length and greater lost body weight also were observed in these *KLPJ*-infused mice compared with the control, although they were more remarkable in mice infused with *E coli* (Figure 3*B* and *C*), indicating that *KLPJ* can directly induce IBD. The increased expression of inflammatory cytokines also was detected (Figure 3*D*). Equal amounts of bacteria also were detected in the colon of infused mice (Figure 3*E*). CD11b^+^ ly6G^+^ inflammatory neutrophils also existed in higher quantities in the mice infused with *KLPJ* (Figure 3*F*). Because isolated *KLPJ* can induce IL18 secretion in gut epithelial cells (Figure 1), which play an important role in the induction of IL22 and IFNγ production, increasing NK cell activity and T-cell proliferation,^24^ we also analyzed T-helper 1 (Th1) cells and NK cells. Both IFNγ^+^Th1 cells and IFNγ^+^ NK cells were increased markedly in the mice infused using *KLPJ* (Figure 3*G* and *H*). IFNγ can promote the generation of inflammatory macrophages.^30^ Increased CD11b^+^Major histocompatibility complex II(MHCII)^+^ly6c^+^ inflammatory macrophages in the *KLPJ-*infused mice also were detected (Figure 3*I*). Thus, the isolated *KLPJ* can induce colitis by itself.

**Figure 4:**
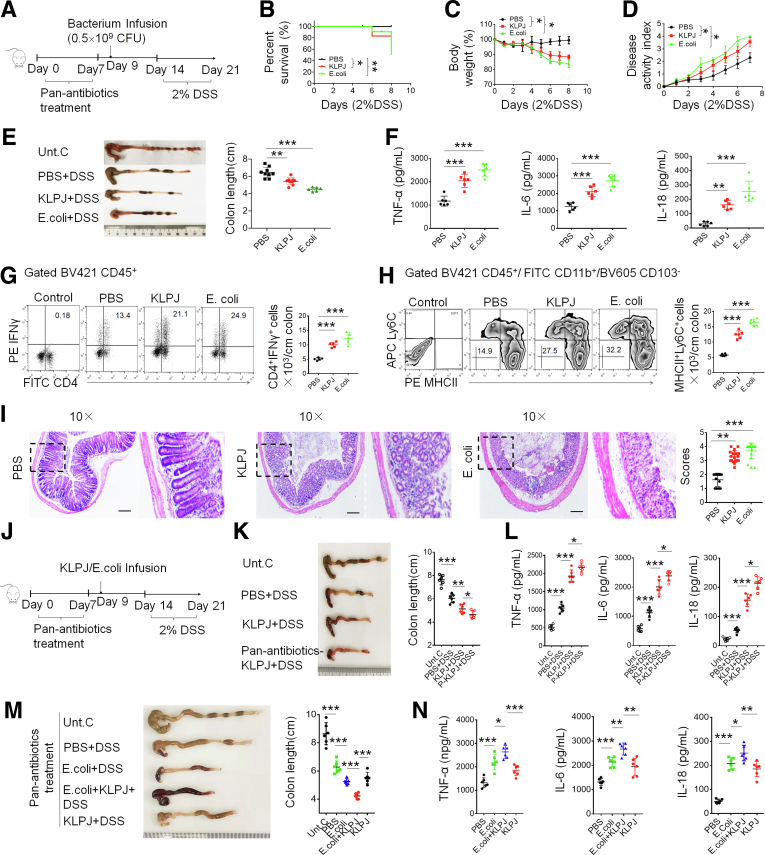
**Isolated *KLPJ* promotes DSS-mediated colitis.** (*A*) Schematic of the experiment for the effects of *KLPJ* on DSS-mediated colitis. (*B*) Survival rate of *KLPJ* or *E coli*–infused mice after DSS (n = 12). (*C*) Body weight of *KLPJ* or *E coli*–infused mice after DSS (n = 12). (*D*) DAI of mice after DSS (n = 12). (*E*) Colon length of mice after DSS (n = 6). (*F*) ELISA of tumor necrosis factor α (TNFα), IL6, and IL18 in the colon tissues of mice (n = 6) after DSS. (*G*) Flow cytometry of CD4^+^IFNγ^+^ cells in the colon tissues of mice after DSS (n = 6). Control indicates IFNγ isotypic control. (*H*) Flow cytometry of CD11b^+^MHCII^+^Ly6C^+^ inflammatory macrophages in the colon tissues of mice after DSS (n = 6). Control indicates isotypic control. (*I*) H&E staining of colon tissues of mice after DSS (3 slides/mouse, n = 6). Mice with (*KLPJ* or *E coli*) or without (PBS) the gavage of *KLPJ* or *E coli*. PBS indicates control. Dashed outline indiates that H/E staining on the right is from this region. *Scale bar*: 40 μm. (*J*) Schematic of the experiment for the effects of *KLPJ* on SPF WT or antibiotics-treated mice in DSS-mediated colitis. (*K*) Colon length of SPF after gavage using *KLPJ* or pan-antibiotics–treated mice after DSS (n = 6). (*L*) ELISA of TNFα, IL6, and IL18 in the colon tissues of SPF mice after gavage by *KLPJ* or pan-antibiotics–treated mice (n = 6) after DSS. (*M*) Colon length of pan-antibiotics–treated mice after gavage by *E coli* and/or *KLPJ* after DSS (n = 6). (*N*) ELISA of TNFα, IL6, and IL18 in the colon tissues of pan-antibiotics–treated mice after gavage by *E coli* and/or *KLPJ* after DSS (n = 6). (*B*) Wilcoxon test; (*C* and *D*) 1-way analysis of variance test; (*E–H* and *K–N*) Student *t* test; (*I*) Mann–Whitney *U* test. ∗*P* < .05, ∗∗*P* < .01, and ∗∗∗*P* < .001. Data are representative of at least 2 experiments. FITC, fluorescein isothiocyanate; Unt. C, untreated healthy mice.

To further investigate the roles of *KLPJ* in colitis, we also used a DSS-mediated colitis model in pan-antibiotics–treated mice (Figure 4*A*). The mice were first infused using *KLPJ*; then, DSS was given 2 days later. We found that the severity of inflammation was increased significantly in the *KLPJ-*infused mice. Compared with the control group, the *KLPJ-*infused mice had greater lost body weight and a higher mortality rate and DAI (Figure 4*B*–*D*). Meanwhile, we also observed a shorter colon in *KLPJ-*infused mice (Figure 4*E*). Inflammatory cytokines also remarkably increased in the colon tissue in DSS-treated colitis mice, which were colonized by *KLPJ* (Figure 4*F*). Inflammatory cells such as CD45^+^CD4^+^IFNγ^+^Th1 helper cells and CD45^+^CD11b^+^MHCII^+^Ly6C^+^ macrophages existed in much higher amounts than in the control group (Figure 4*G* and *H*). H&E staining showed that the colon suffered more severe injury. The epithelial layer also was incomplete with the infection of *KLPJ* (Figure 4*I*). Most IBD patients carry heavy *E coli* loads, whereas *KLP* are less frequent. We do not know whether *KLP* would have a limited effect on colitis in the presence of *E coli.* Thus, it would be important to know if a similar degree of DSS colitis would happen in SPF mice not receiving antibiotics, but given DSS. To test this, SPF and pan-antibiotics–treated mice were infused using *KLPJ*, and then DSS. We indeed observed limited effects of *KLPJ* in the presence of other bacteria (Figure 4*J–L*). This may be derived from protective roles of gut microbiota.^31^ However, in pan-antibiotics–treated SPF mice, *KLPJ* promoted *E coli*–mediated colitis after DSS (Figure 4*M* and *N*). Taken together, these results suggest that the *KLPJ* isolated from UC patients can induce and promote colitis.

### *K pneumoniae*–Mediated Colitis Occurs Through IL18 in Gut Epithelial Cells

**Figure 5:**
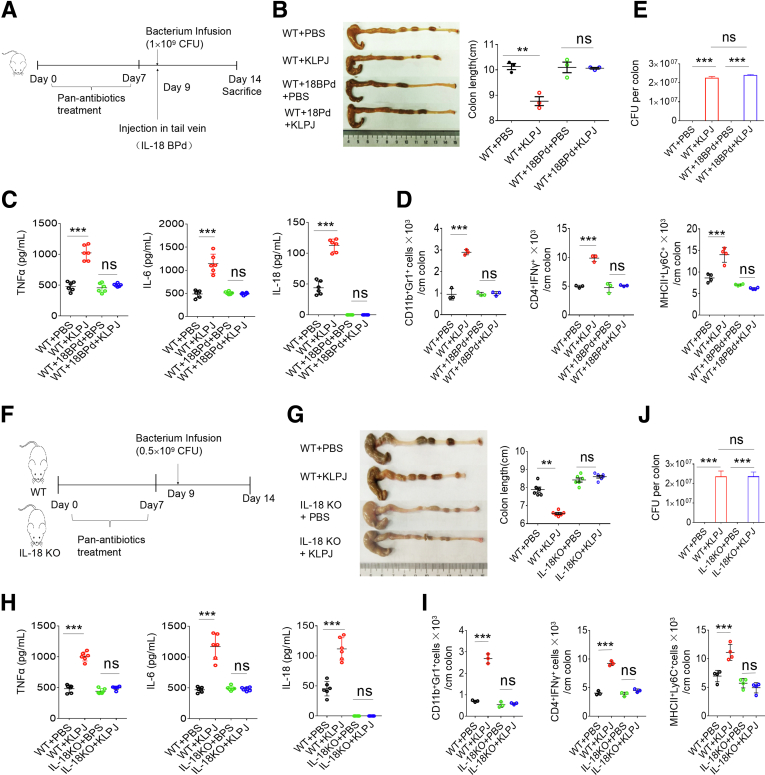
***KLPJ*-mediated colitis depends on IL18.** (*A*) Schematic of the experiment for the effects of IL18 blocking reagent (IL18 Binding protein isoform d) on *KLPJ-*mediated colitis. (*B*) Colon lengths in mice (n = 6). (*C*) ELISA of tumor necrosis factor α (TNFα), IL6, and IL18 in the colon tissues in mice (n = 6). (*D*) Flow cytometry of CD11b^+^Gr1^+^, CD4^+^IFNγ^+^, and MHCII^+^Ly6C^+^ cells in mice (mixed sample per 2 mice, n = 6). (*E*) CFU of *KLPJ* in the colon contents of mice. For CFU in bacteria-infused mice, 10^9^ bacteria were orally infused and then CFU were counted after 7 days. (*F*) Schematic of the experiment for *KLPJ*-mediated colitis in WT and *IL18* KO mice. (*G*) Colon lengths of WT and *IL18* KO mice (n = 6). (*H*) ELISA of IL18 in the colon tissues in WT and *IL18* KO mice (n = 6). (*I*) Flow cytometry of immune cells in the colon tissues of WT and *IL18* KO mice (mixed sample per 2 mice, n = 6). (*J*) CFU of *KLPJ* in the colon contents of WT and *IL18* KO mice. For CFU in bacteria-infused mice, 10^9^ bacteria were orally infused and then CFU were counted after 7 days. (*B–F*) Mice with (WT+18BPd+PBS or WT+18BPd+KLPJ) or without (WT+BPS or WT+KLPJ) IL18 blocking reagent (IL18BPd) and with (WT+18BPd+KLPJ or WT+KLPJ) or without (WT+18BPd+PBS or WT+BPS) *KLPJ* (KLPJ); (*H–L*) WT and *IL18* KO mice with (WT+KLPJ or IL18KO+KLPJ) or without (WT+BPS or IL18KO+BPS) gavage of *KLPJ*. Student *t* test. ∗∗*P* < .01 and ∗∗∗*P* < .001. Data are representative of at least 2 experiments.

IL18 in the gut epithelial cells is a key determining factor in IBD.^17^^,^^18^ The secretion of mature IL18 in the colon after exposure to *KLPJ* was much higher than in the control group (Figure 1, Figure 2, Figure 3, Figure 4), suggesting that *KLPJ*-mediated colitis occurs through IL18. To investigate the involvement of IL18, we first used an IL18-blocking regent (IL18 Binding protein isoform d) to observe the effects on *KLPJ*-mediated colitis (Figure 5*A*). Data showed that blocking IL18 in the SPF mice significantly reduced inflammation (Figure 5*B*–*E*). To confirm this, we also used *IL18* knockout (KO) and their littermates to observe the effects of *IL18* deficiency on *KLPJ*-mediated colitis (Figure 5*F*). A marked difference existed in the *KLPJ-*mediated colitis between the WT and *IL18 KO* mice. Unlike the wild-type (WT) mice, the *KLPJ-*infused *IL18* KO mice did not show a significant difference in colon length, inflammatory cytokines, and inflammatory immune cells compared with the *IL18* KO mice without gavage of *KLPJ* (Figure 5*G*–*I*), indicating that IL18 is involved in *KLPJ-*mediated colitis. The equal amount of *KLPJ* in the colon of the infused mice is shown in Figure 5*J*.

**Figure 6:**
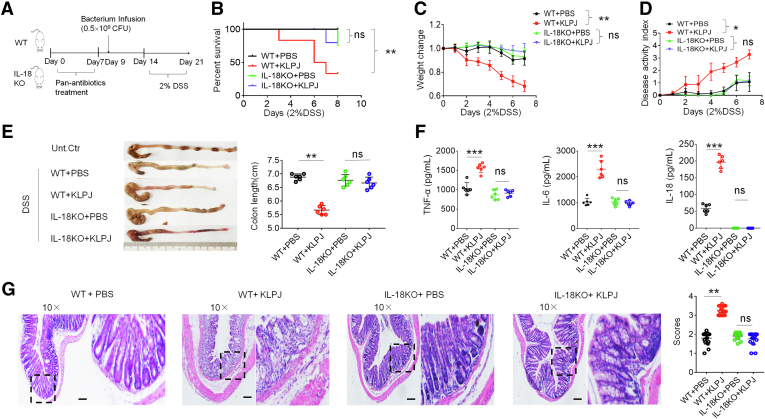
***KLPJ*-mediated colitis after DSS is through IL18.** (*A*) Schematic of the experiment for the effects of *KLPJ* on DSS-mediated colitis in IL18 KO mice. (*B*) Survival rate of WT and their littermate IL18 KO mice (n = 12) after DSS. (*C*) Body weight changes of WT and their littermate IL18 KO mice (n = 12) after DSS. (*D*) Disease activity index of WT and their littermate IL18 KO mice (n = 12) after DSS. (*E*) Colon length of WT and their littermate IL18 KO mice (n = 6) after DSS. (*F*) ELISA of tumor necrosis factor α (TNFα), IL6, and IL18 in the mixed samples of colon tissues of WT and *IL18* KO mice (n = 6) after DSS. (*G*) H&E staining of colon tissues of WT and their littermate IL18 KO mice (3 slides/mouse, n = 6) after DSS. *Scale bars*: 40 μm. WT and IL18 KO mice with (WT+KLPJ or IL18KO+KLPJ) or without (WT+PBS or IL18 KO+PBS) gavage of *KLPJ*. PBS indicates control. (*B*) Wilcoxon test; (*C* and *D*) 1-way analysis of variance test; (*E* and *F*) Student *t* test; and (*G*) Mann–Whitney *U* test. Dashed outline indiactes that H&E staining on the right is from this region. ∗*P* < .05, ∗∗*P* < .01, and ∗∗∗*P* < .001. Data are representative of at least 2 experiments. Unt. Ctr, untreated control.

We also observed the effects of *KLPJ* on DSS-mediated colitis in *IL18* KO mice (Figure 6*A*). The mice were first infused using *KLPJ*; then, DSS was given 2 days later. There also was no difference in body weight, mortality, DAI, and inflammatory cytokines in the IL18 KO mice with or without gavage of *KLPJ* (Figure 6*B*–*F*). H&E staining also did not show more severe injury in IL18 KO mice infused with *KLPJ* compared with the control group (Figure 6*G*). All of these results suggest that *KLPJ-*mediated colitis depends on IL18.

**Figure 7:**
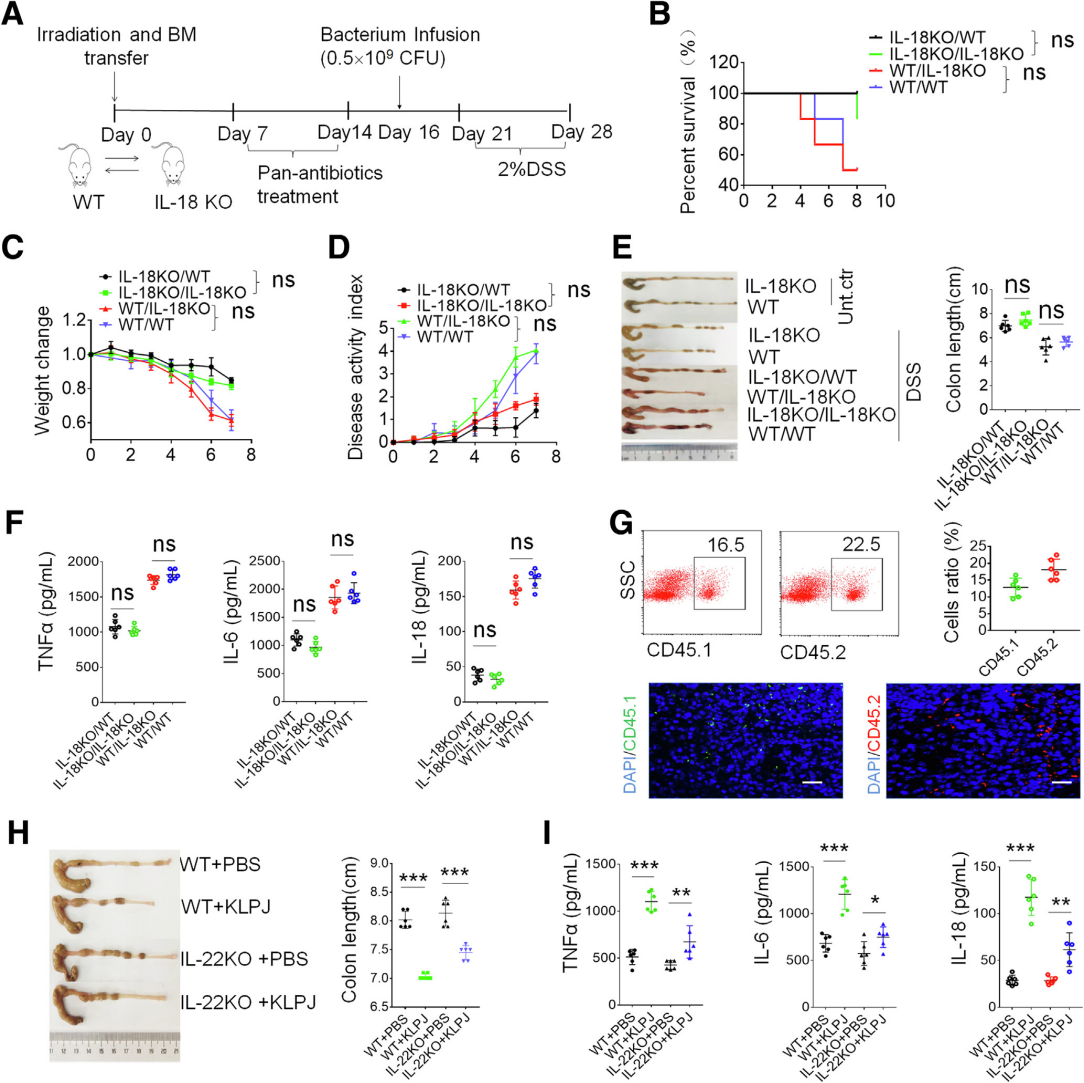
**Transplantation of WT or *IL18* KO bone marrow cells does not affect *KLPJ-*mediated colitis.** (*A*) Schematic of the effects of bone marrow cell (BMC) transplantation on *KLPJ-*mediated colitis. (*B*) Survival rates in BMC transplanted WT or *IL18* KO mice after DSS (n = 12). (*C*) Body weight changes in BMC transplanted WT or *IL18* KO mice after DSS (n = 12). (*D*) Disease activity index in BMC transplanted WT or *IL18* KO mice after DSS (n = 12). (*E*) Colon length in BMC transplanted WT or *IL18* KO mice after DSS (n = 6). (*F*) ELISA of tumor necrosis factor α (TNFα), IL6, and IL18 in the colon tissues in BMC transplanted WT or *IL18* KO mice (n = 6) after DSS. (*G*) Flow cytometry (*upper*) and immunostaining (*lower*) of CD45.1 or CD45.2 cells in the spleens of IL18KO/WT (CD45.1) or WT/IL18KO (CD45.2) mice (n = 6) using APC-labeled CD45.1 or PE-labeled CD45.2. *Scale bar*: 40 μm. (*H*) Colon length in WT and *IL22* KO mice (n = 6). (*I*) ELISA of IL18 in the colon tissues in WT and *IL22* KO mice (n = 6). IL18KO/WT, BMCs from CD45.1 WT into irradiated CD45.2 *IL18* KO mice; WT/IL18KO, BMCs from CD45.2 *IL18* KO mice into irradiated CD45.1 WT mice; IL18KO/IL18KO, BMCs from *IL18* KO mice into irradiated *IL18* KO mice; WT/WT, BMCs from WT mice into irradiated WT mice. BMC transplanted mice then were infused with *KLPJ*, and treated using DSS. (*B*) Wilcoxon test; (*C* and *D*) 1-way analysis of variance test; and (*E–I*) Student *t* test. ∗*P* < .05, ∗∗*P* < .01, and ∗∗∗*P* < .001. Data are representative of at least 2 experiments. SSC, SideScatter; Unt. ctr., untreated control healthy mice.

Although IL18 is produced mainly by gut epithelial cells, gut macrophages also produce IL18.^32^^,^^33^ To illustrate that gut epithelial cell–derived IL18 plays a main role in *KLPJ-*mediated colitis, we performed bone marrow transplant experiments. Transplantation of bone marrow cells from *IL18* KO into CD45.1^+^ WT or WT into CD45.2^+^ *IL18* KO mice did not produce significantly different effects on *KLPJ-*mediated colitis (Figure 7*A–G*), indicating that IL18 in gut epithelial cells plays a main role in *KLPJ-*mediated colitis. The IL18 generated by the gut epithelial cells could drive Group 3 innate lymphoid cell proliferation and promotes IL22 production.^34^^,^^35^ *KLPJ* also was infused into pan-antibiotics–treated *IL22* KO mice. Here, there also were differences in colon lengths and inflammatory cytokines in the *KLPJ-*infused *IL22* KO mice compared with the uninfused *IL22* KO mice, although this difference was less than those in WT with or without gavage (Figure 7*H* and *I*), suggesting that IL22 also is partly involved in *KLPJ-*mediated colitis. Taken together, *KLPJ*-mediated colitis mainly depends on IL18 derived from colon epithelial cells.

### *K pneumoniae*–Mediated Colitis Occurs Through Caspase-1/11 but Not NLR Family Caspase Recruitment Domain Containing 4 or NOD-like receptor thermal protein domain associated protein 3 (NLRP3)

**Figure 8:**
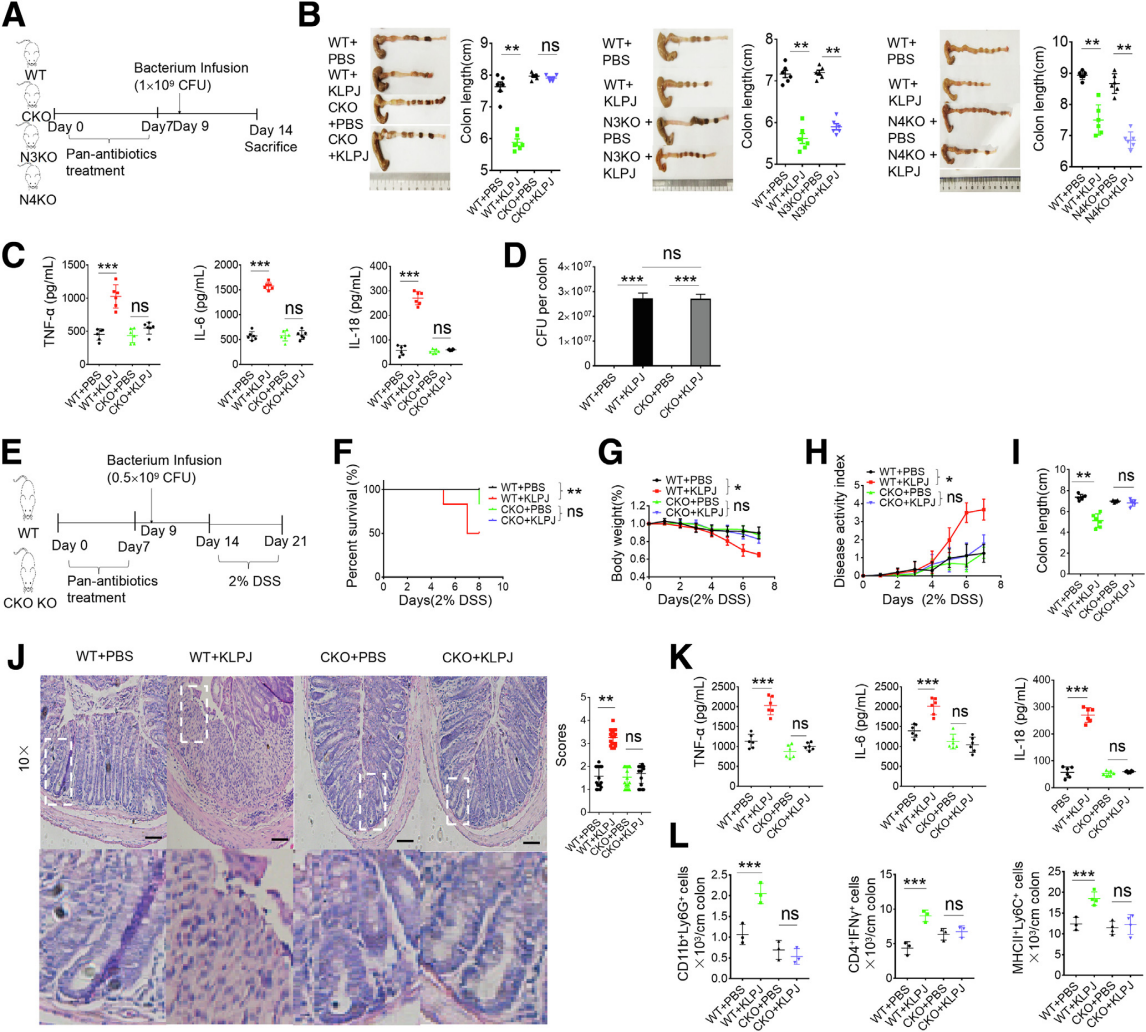
***KLPJ*-mediated colitis depends on caspase-1/11, but not NLRP3 or NLRC4.** (*A*) Schematic of the experiment for the effects of *caspase-1/11* KO (CKO), *NLRP3* KO (N3KO), and *NLRC4* KO (N4KO) on *KLPJ-*mediated colitis. (*B*) Colon length in *caspase-1/11* KO (CKO+KLPJ), *NLRP3* KO (N3KO+KLPJ), and *NLRC4* KO (N4KO+KLPJ) or WT (WT+KLPJ) mice after infusing *KLPJ* (n = 6). WT+PBS, CKO+PBS, N3KO+PBS and N4KO+PBS, controls. (*C*) ELISA of tumor necrosis factor α (TNFα), IL6, and IL18 in the mixed samples of colon tissues of WT and *caspase-1/11* KO mice (n = 6). (*D*) CFUs of *KLPJ* in colon contents of mice. Bacteria (10^9^) were orally infused and then CFU were counted after 7 days. (*E*) Schematic of the effects of *KLPJ* on the DSS-mediated colitis in CKO mice. (*F*) Survival rates of WT and their littermate *caspase-1/11* KO mice (n = 12) after DSS. (*G*) Body weight changes of WT and their littermate *caspase-1/11* KO mice (n = 12) after DSS. (*H*) DAI of WT and their littermate *caspase-1/11* KO mice (n = 12) after DSS. (*I*) Colon length of WT and their littermate *caspase-1/11* KO mice (n = 6) after DSS. (*J*) H&E staining of colon tissues of WT and their littermate *caspase 1/11* KO mice (3 slides/mouse, n = 6) after DSS. *Scale bars*: 40 μm. (*K*) ELISA of TNFα, IL6, and IL18 in the mixed samples of colon tissues of WT and their littermate *caspase-1/11* KO mice (n = 6) after DSS. (*L*) Flow cytometry of CD45^+^CD11b^+^Ly6G^+^, CD45^+^CD11b^+^CD103^-^MHCII^+^Ly6C^+^, or CD45^+^CD4^+^IFNγ^+^ immune cells in the colon tissues of WT and their littermate *caspase-1/11* KO mice (mixed sample per 2 mice, n = 6) after DSS. WT and CKO mice with (WT+KLPJ or CKO+KLPJ) or without (WT+PBS or CKO+ PBS) gavage of *KLPJ*. (*F*) Wilcoxon test; (*G* and *H*) 1-way analysis of variance test; (*B*–*D*, *I*, *K*, and *L*) Student *t* test; and (*J*) Mann–Whitney *U* test. ∗*P* < .05, ∗∗*P* < .01, and ∗∗∗*P* < .001. Data are representative of at least 2 experiments.

A common manifestation in individuals infected by *KLP* is pneumonia.^5^^,^^6^ Previous studies have shown that NLRP3, NLR family CARD domain containing 4 (NLRC4), or caspase-11 was required for *KLP* infection in the lungs.^36^^,^^37^ Gut epithelial cells have shown expression of an array of inflammasome components including NLRC4, caspase-1, caspase-4/5 (human)/caspase-11 (mouse), caspase-8, apoptosis-associated speck-like protein containing a card, and NLRP6/3,^38^ which finally cause mature IL18 production.^39^ *KLPJ* belongs to gram-negative bacteria, which potentially can produce lipopolysaccharide (LPS).^15^ Caspase-11 in mice and caspase-4/5 in human beings can function as intracellular receptors for LPS.^39^^,^^40^ Thus, we used *caspase-1/11* KO mice, in which caspase-11 was deleted together with caspase-1 during generation^39^ to investigate the role of caspase-1/11 in *KLPJ-*mediated colitis. We found that the gavage of *KLPJ* did not cause colitis in *caspase-1/11* KO mice and *KLPJ* also did not promote DSS-mediated colitis in these *caspase-1/11* KO mice (Figure 8); whereas their littermates with or without *KLPJ* gavage were markedly different not only in non–DSS-treated mice (Figure 8*A*–*D*) but also in DSS-mediated colitis (Figure 8*E–L*). Caspase-11 activation can be triggered by LPS binding via the CARD domain, which leads to the activation of NLRP3 and pyroptosis.^40^ We next also addressed the function of NLRP3 in *KLPJ-*mediated colitis. Similar to WT mice, *NLRP3* KO mice also showed marked colitis after gavage of *KLPJ* (Figure 8*B*), suggesting that NLRP3 is not involved significantly in *KLPJ*-mediated colitis. NLRC4 is essential for the *KLP*-induced production of IL1β, IL17A, and neutrophil chemoattractants in the lungs.^36^ Certain strains of KLP have been shown to possess flagella,^41^ and flagella can activate NLRC4.^42^ However, the data did not show the involvement of NLRC4 in *KLPJ-*mediated colitis (Figure 8*B*). Notably, there was an equal number of *KLPJ* in all experimental mice after infusing them with *KLPJ* for 7 days (Figure 8*D*). Taken together, *KLPJ-*mediated colitis occurs through caspase-1/11, but not NLRC4 or NLRP3.

### KLPJ-Mediated Colitis Occurs Through the Binding of LPS With Caspase-11

**Figure 9:**
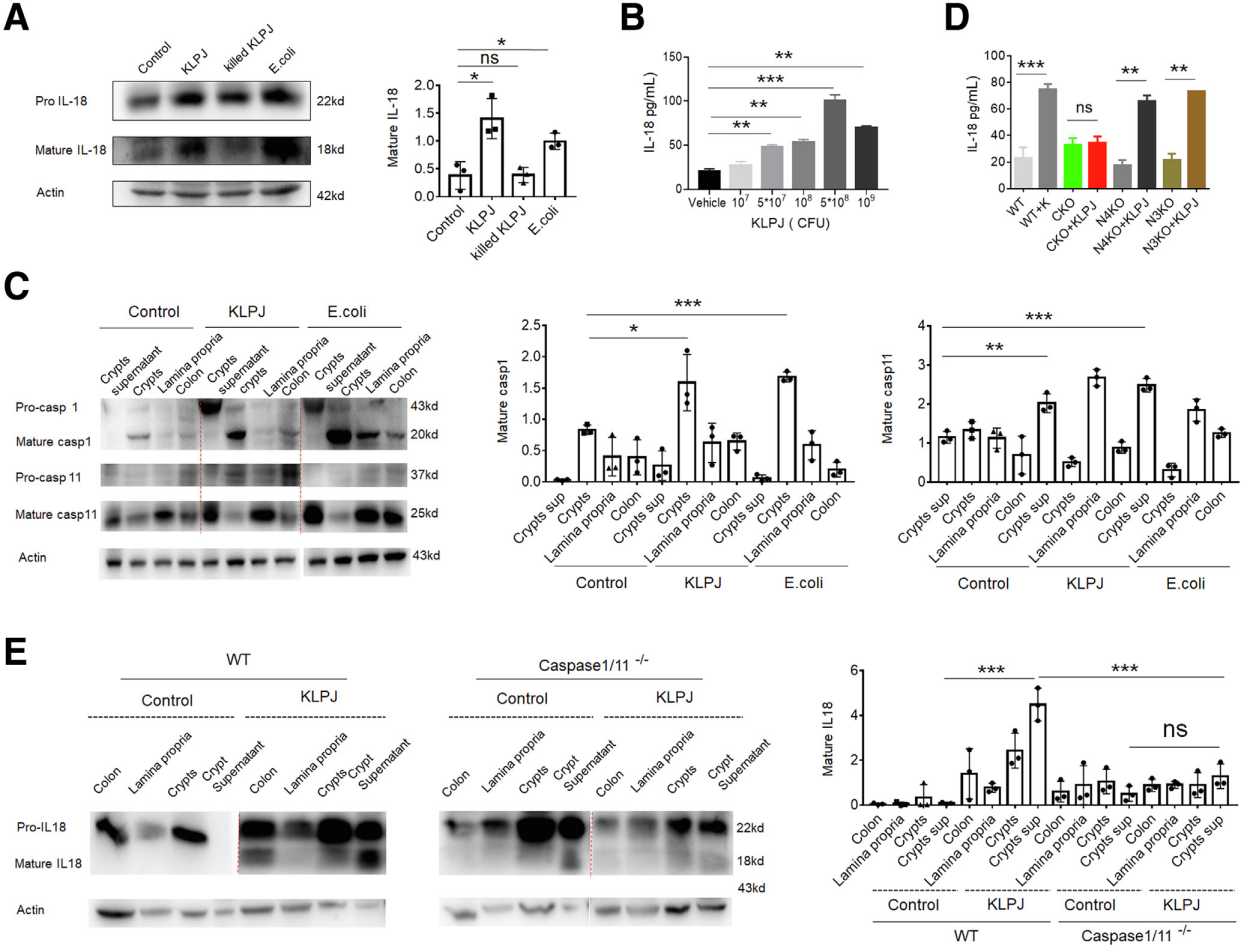
***KLPJ*-mediated IL18 in colon epithelial cells depends on caspase-1/11.** (*A*) Immunoblotting of pro-mature and mature IL18 in the colon epithelial cells after exposure to alive *KLPJ* (KLPJ), killed *KLPJ* (killed KLPJ), and *E coli*. Control indicates PBS. (*B*) ELISA of IL18 in the supernatants of the colon after exposure to different concentrations of *KLPJ*. (*C*) Immunoblotting of pro- and mature caspase-1 and caspase-11 in the crypt supernatants, crypts, lamina propria, and colon after exposure to *KLPJ*. (*D*) ELISA of IL18 in the supernatants of the colon of WT, *caspase-1/11* KO, *NLRP3* KO, and *NLRC4* KO mice after exposure to *KLPJ*. The colons were isolated from different mice, and then exposed to *KLPJ*. (*E*) Immunoblotting of pro- and mature IL18 in the crypt supernatants, crypts, lamina propria, and colon of WT *caspase-1/11* KO mice after exposure to *KLPJ*. Actin was used as a loading control. Student *t* test. ∗*P* < .05, ∗∗*P* < .01, and ∗∗∗*P* < .001. sup, supernatatnts.

IL18 secretion in epithelial cells is controlled by inflammasomes, which include the NLR and caspase families.^21^^,^^43^ We determined how *KLPJ* induced the generation of mature IL18. We used different concentrations of *KLPJ* to stimulate the colon, isolated colon epithelial cells for Western blot, and collected the colon contents for mature IL18. Consistent with Figure 1, *KLPJ* induced the generation of mature IL18 (Figure 9*A* and *B*). Mature IL18 requires coordinated inflammasome activation of the cysteine protease caspase-1 to cleave pro-IL18 into a functional mature bioactive cytokine.^22^^,^^23^ *KLPJ* also could activate pre–caspase-1 to mature p20 caspase-1 (Figure 9*C*). To further study the role of caspase-11, NLRP3, and NLRC4 in *KLPJ-*mediated colitis, the colons from *caspase-1/11* KO, *NLRP3* KO, and *NLRC4* KO mice were stimulated with *KLPJ* ex vivo. Compared with the WT mice, mature IL18 in the supernatants of the colons decreased significantly in the *caspase-11/1* KO mice but not in those of *NLRP3* KO and *NLRC4* KO mice after exposure to *KLPJ*, indicating the necessity of caspase-1/11 in *KLPJ*-induced IL18 secretion by colon epithelial cells (Figure 9*D* and *E*). Taken together, *KLPJ*-mediated generation of mature IL18 is dependent on caspase-1/11, but not NLRP3 or NLRP4.

**Figure 10:**
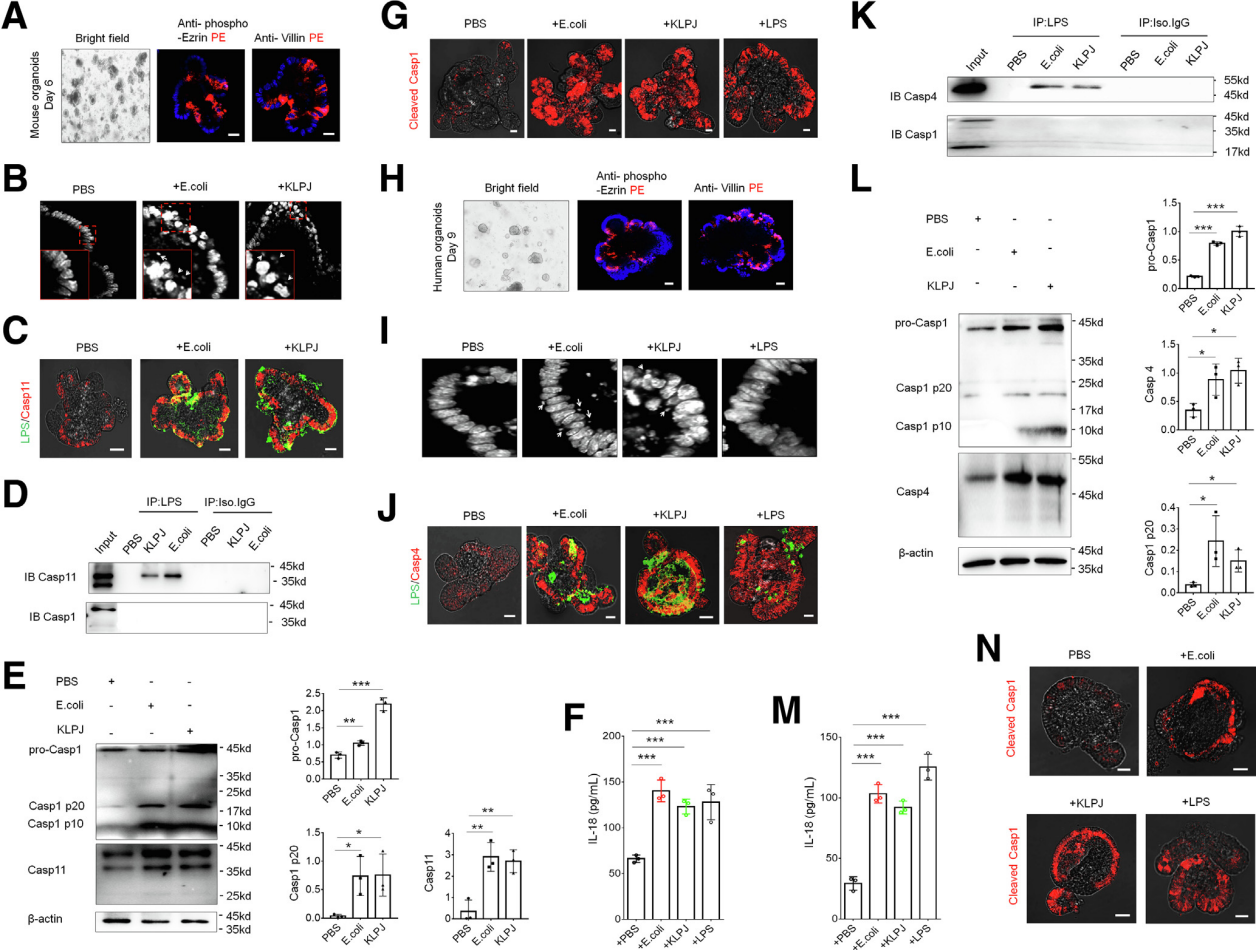
***KLPJ*-mediated colitis is through binding of LPS from *KLPJ* with caspase-11 in the colonic epithelial cells.** (*A*) Staining of mouse gut organoids (6 days) using anti–phospho-ezrin or villin. (*B*) *E coli* and *KLPJ* under a light microscope in gut organoids. *Arrow* indicates *E coli* or *KLPJ*. (*C*) Fluorescence microscope of the binding of LPS and caspase-11 in gut organoids. (*D*) Immunoprecipitation using biotin-labeled LPS, and then immunoblotting using anti–caspase-11 or anti–caspase-1 antibody in the lyses of *KLPJ*–infected gut organoids. (*E*) Immunoblotting of pro-caspase 11 and mature caspase-11, and pro- and mature caspase-1 in the lyses of *KLPJ* or *E coli*–infected gut organoids. (*F*) ELISA of IL18 in the supernatants of *KLPJ*, *E coli*–infected gut organoids. LPS indicates control. (*G*) Staining of cleaved caspase-1 (cleaved casp-1) in *KLPJ* or *E coli*–infected gut organoid. LPS indicates control. (*H*) Staining of human gut organoids (9 days) using anti–phospho-ezrin or villin. (*I*) *E coli* and *KLPJ* under a light microscope in human colon organoids. *Arrows* indicates *E coli* or *KLPJ*. LPS indicates control. (*J*) Fluorescence microscope of the binding of LPS and caspase 4 in human colon organoid. LPS indicates control. (*K*) Immunoprecipitation using biotin-labeled LPS, and then immunoblotting using anti–caspase-4 or anti–caspase-1 antibody in the lyses of *KLPJ*–infected human colon organoid. (*L*) Immunoblotting of mature caspase-4 and pro- and mature caspase-1 in the lyses of *KLPJ* or *E coli*–infected human colon organoids. (*M*) ELISA of IL18 in the supernatant of *KLPJ* or *E coli*–infected human colon organoids. (*N*) Staining of cleaved caspase-1 (cleaved casp-1) in *KLPJ* or *E coli*–infected human colon organoids. For the entry and organoid immunofluorescence staining of *KLPJ*, microinjections were performed. For stimulation of *KLPJ* on gut organoids, the suspension of gut organoids was prepared. Student *t* test. *Scale bars*: 40 μm. ∗*P* < .05, ∗∗*P* < .01, and ∗∗∗*P* < .001. IB, immunoblotting; IP, immunoprecipitation; Iso,Isotype control.

Multiple Gram negative bacteria such as *E coli* could activate caspase-11 through LPS.^39^ *KLPJ-*mediated mature IL18 generation in colonic epithelial cells can be through caspase-1/11, implying that LPS from *KLPJ* can activate caspase-11. To investigate this, we cultured in vitro colon organoids and then exposed these colon organoids to *KLPJ*. Gut organoids were cultured in mouse or human organoid growth medium, which could induce differentiation of gut organoids (Figure 10*A* and *H*).

We next detected the binding of LPS from *KLPJ* with caspase-11. To test this*,* we performed organoid microinjections. Microscope and pull-down studies not only showed the existence of *KLPJ* in the cells, but also indicated the binding of LPS with caspase-11 (Figure 10*B*–*D*). Meanwhile, we also detected the fragments of caspase-1, which can be degraded by activated caspase-11.^39^ More fragments were found in the *KLPJ-*infected cells (Figure 10*E*). For the stimulation of *KLPJ* on gut organoids, we prepared the suspensions of gut organoids. IL18 levels in the supernatants of gut organoids after exposure to *KLPJ* were higher compared with the control group (Figure 10*F*). Caspase-11–mediated activation of caspase-11 also caused cellular pyroptosis.^44^ More pyroptotic cells were observed in the *KLPJ-*infected gut organoids (Figure 10*G*). Similar results also were found in the human colon organoids (Figure 10*I*–*N*). Thus, these data indicate that KLPJ can colonize the colon, activate caspase-11, and contribute to intestinal inflammation.

## Discussion

In this study, we isolated a strain of *KLP*, named *KLPJ*, from the colon tissues of patients with UC. We found that this isolated *KLPJ* induced colitis and promoted DSS-mediated colitis. We showed that *KLPJ*-mediated colitis occurred through the caspase-11–mediated release of mature IL18 in the gut epithelial cells, which plays an important role in the induction of IFNγ production, increasing NK cell activity and T-cell proliferation. This strain of *KLPJ* released LPS to combine with caspase-11 and activate it. Thus, our study uncovered the interactions among *KLPJ*, intestinal epithelial cells, and gut immune cells, providing new insights for the role of *KLP* in the development of gut diseases such as IBD.

We found that *KLPJ* induced colitis and promoted DSS-mediated colitis through caspase-11. Previous studies have shown that *KLP* could invade human epithelial cells and stably colonize and persist in the gastrointestinal tract of mice.^45^, ^46^, ^47^ Oral administration of *KLP* also increased the expression of cyclooxygenase-2, IL1β, IL6, and tumor necrosis factor α, and the activation of nuclear factor-κB in the colon.^8^ *KLP* J2H7 from saliva strongly drove Th1 cell induction and inflammation in the intestine, especially in *IL10* KO gene susceptible mice.^48^ Other investigators also found that caspase-11 contributed to the pulmonary host defense against *KLP* and local activation of coagulation.^49^ *Caspase-11* deficiency impaired neutrophil recruitment and bacterial clearance in the early stage of pulmonary *KLP* infection.^37^

We showed that *KLPJ*-mediated activation of the caspase-1/11 pathway was through the interaction of LPS from *KLPJ* with caspase-11. Caspase-11 as a noncanonical inflammasome can function as an intracellular receptor for LPS and is activated by direct binding to LPS.^39^ For noncanonical inflammasome activation, bacterial products such as LPS are translocated into the host cytosol.^43^ Cytoplasmic LPS binds directly to the CARD motif of caspase-11 with its lipid A moiety, leading to oligomerization of caspase-11 to activate noncanonical inflammasomes. Active caspase-11 also cleaves Gasdermin D. to release its N-terminal domain, which subsequently is inserted into the plasma membrane to form membrane pores.^40^^,^^50^ In human beings, LPS can mediate noncanonical (caspase-4/11–dependent) inflammasome activation, when mammalian immune cells are challenged with intracellular bacteria including *Shigella flexneri*, *Salmonella Typhimurium*, *Legionella pneumophila*, *Francisella novicida*, several *Burkholderia* species, and *Chlamydia trachomatis*, as well as extracellular bacteria such as enterohemorrhagic *E coli*, *Citrobacter rodentium*, and *Yersinia pseudotuberculosis.*^39^

The most common manifestations in individuals infected by *KLP* are pneumonia and urinary tract and wound infections.^5^^,^^6^ Previous studies have shown that NLRP3, NLRC4, or caspase-11 are required during *KLP* invasion in the lungs, indicating their important role in *KLP* infection.^36^^,^^37^ NLRC4 is essential for *KLP*-induced production of IL1β, IL17A, and neutrophil chemoattractants in the lungs.^36^ Transmission electron microscopy with Ryu staining also showed that *KLP* JBUAP021 expresses polar flagella.^41^ However, we did not find the involvement of NLRC4 in *KLPJ-*mediated colitis. Other studies have shown that NLRP3 inflammasomes were involved in regulating colonic immune homeostasis; Huber et al^51^ showed that both NLRP3 and NLRP6 inflammasomes mediated production of IL18. However, we did not find that NLRP3 participated in the *KLPJ-*mediated colitis and secretion of *KLPJ-*mediated IL18. Other investigators also showed that caspase-4/11 in intestine epithelial cells could act independently of NLRP3 to directly process IL18 and induce pyroptosis during *Salmonella* infection.^43^

## Materials and Methods

The reagents and oligomers used in this study are listed in Table 1.

**Table 1:** Reagents and Oligoes Used in This Study

Reagent or resource (clone name)	Source	Identifier
Antibodies for immunoblotting and immunostaining		
HRP-goat anti-mouse IgG (H+L)	zsbio	Cat: ZB2305
Anti-mouse β-actin (C4)	Santa Cruz Biotechnology	Cat: sc-47778 RRID:AB_626632
Anti-mouse IL18	Abcam	Cat: ab71495 RRID:AB_1209302
Anti-mouse CD11b (1B6e)	Santa Cruz Biotechnology	Cat: sc-21744 RRID:AB_626882
Anti-mouse/human caspase-1	Proteintech	Cat: 22915-1-Ap RRID:AB_2876874
Anti-mouse caspase-11 (17D9)	Cell Signaling Technology	Cat: 14340
Anti-LPS	Abcam	Cat: ab35654 RRID:AB_732222
Anti-human caspase-4 (4B9)	Santa Cruz Biotechnology	Cat: sc-56056 RRID:AB_781828
Anti-mouse/human NLRP3 (EPR23094-1)	Abcam	Cat: ab263899 RRID:AB_2889890
Anti-mouse/human NLRC4	Invitrogen	Cat: PA5-88997 RRID:AB_2805284
Anti-mouse IL1β	ABclonal Biotechnology	Cat: A11369 RRID:AB_2758528
Anti-mouse CD45.1 (A20)	Abcam	Cat: ab25078 RRID:AB_448593
Anti-mouse CD45.2 (104)	Invitrogen	Cat: 14-0454-85 RRID:AB_467262
Anti–phospho-ezrin	Affinity	Cat: AF3172 RRID:AB_2834604
Anti-villin	Proteintech	Cat: 16488-1-AP RRID:AB_2215975
Antibodies for flow cytometry		
7-AAD	BioLegend	Cat: 640926
Brilliant Violet 421 anti-mouse CD45 (30-F11)	BioLegend	Cat: 103133 RRID:AB_10899570
PE anti-mouse MHCII (M5/114.15.2)	BioLegend	Cat: 107608 RRID:AB_313323
APC anti-mouse Ly6C (HK1.4)	BioLegend	Cat: 128016 RRID:AB_1732076
FITC anti-mouse CD4 (RM4-5)	Thermo Fisher Scientific	Cat: 11-0042-85 RRID:AB_464897
PE anti-mouse IFNγ (XGM1.2)	Thermo Fisher Scientific	Cat: 25-7311-82 RRID:AB_469680
FITC anti-mouse NKp46 (29A1.4 )	BioLegend	Cat: 137606 RRID:AB_2298210
FITC anti-mouse F4/80 (BM8)	BioLegend	Cat: 123108 RRID:AB_893502
FITC anti-mouse CD11b (M1/70)	Thermo Fisher Scientific	Cat: 11-0112-82 RRID:AB_464935
APC anti-mouse TNFα (MP6-XT22)	Thermo Fisher Scientific	Cat: 17-7321-82 RRID:AB_469508
PE anti-mouse Ly6G (1A8)	BD Bioscience	Cat: 551461 RRID:AB_394208
FITC anti-mouse Ly6C (AL-21)	BD Bioscience	Cat: 553104 RRID:AB_394628
APC anti-mouse CD45 (30-F11)	BioLegend	Cat: 103112 RRID:AB_312977
Brilliant Violet 605 anti-mouse CD103 (2E7)	BioLegend	Cat: 121433 RRID: AB_2629724
Oligoes for detection of KLPJ		
16s 27F	BGI	5’- AGAGTTTGATCCTGGCTCAG-3’
16s 1492R	BGI	5’- GGTTACCTTGTTACGACTT-3’
Probe		
Kp-JCM 1662	Synbio Technologies	Cy3- 5’- CCTACACACCAGCGTGCC-3’
Control probe	Synbio Technologies	Cy3-5’- GCACAGTGGACGTCTCAC-3’
Commercial assays		
Mouse IL18 ELISA kit	Elabscience	Cat: E-EL-M0730c
Mouse IL1β ELISA kit	Elabscience	Cat: E-EL-M0037c
Mouse TNF-α ELISA kit	Elabscience	Cat: E-MSEL-M0002
Mouse IL6 ELISA kit	Elabscience	Cat: E-EL-M0044c
Human IL18 ELISA kit	Elabscience	Cat: E-EL-H0253c
QIAquick PCR purification kit	Qiagen	Cat: 28104
QuantiTect SYBR Green PCR Master Mix	Qiagen	Cat: 208052
Cell stimulation cocktail	eBioscience	Cat: 00-4975-03
Permeabilization buffer	Thermo Fisher	Cat: 00-8333-56
ECL chemiluminescence	Absin	Cat: abs920
Protease inhibitor cocktail	Sigma-Aldrich	Cat: P8340
Chemicals		
DSS	Mpbio	Cat: 160110
Ampicillin	Sigma-Aldrich	Cat: BP021
Vancomycin	Sigma-Aldrich	Cat: V2002
Neomycin sulfate	Sigma-Aldrich	Cat: N6386
Metronidazole	Sigma-Aldrich	Cat: M3761
TRIzol	Life Technologies	Cat: 15596026
FBS	Gibco	Cat: 10099141
Collagenase IV	Sigma-Aldrich	Cat: C5138
DNase I	Solarbio	Cat: D8071
DMEM	Gibco	Cat: 11965118
HBSS	Gibco	Cat: 14170161
Percoll	Solarbio	Cat: P8370
PMA	Sigma-Aldrich	Cat: 79346
GolgiStop	BD Biosciences	Cat: 554724
ATP	Sigma-Aldrich	Cat: FLAAS
EDTA	Sigma-Aldrich	Cat: 798681
Thioglycollate	Millipore	Cat: 70157
Pierce Protein A/G magnetic beads	Thermo Fisher	Cat: 88803
Gentle Cell Dissociation Reagent	STEMCELL	Cat: 07174
DAPI	SouthernBiotech	Cat: 0100-20
Y-27632	MCE	Cat: HY-10583
IntestiCult OGM Mouse Kit	STEMCELL	Cat: 06005
IntestiCult OGM Human Kit	STEMCELL	Cat: 06010
Cell strainer	Biosharp	Cat: BS-70-XBS
Matrigel MariX	Corning	Cat: 356231
DMEM/F12	STEMCELL	Cat: 36254

AAD, Aminoactinomycin D; ATP, adenosine triphosphate; ECL, Enhanced chemilluminescience; FITC, fluorescein isothiocyanate; HBSS, Hank’s balanced salt solution; HRP, horseradish peroxidase; NLRC4, NLR family CARD domain containing 4; PCR, polymerase chain reaction; PMA, phorbol 12-myristate 13-acetate; RRID: AB, Research Resource Identifiers-Allen-Bradley; TNFα, tumor necrosis factor α.

### Mice

Four- to 6-week-old male or female C57BL/6 mice were obtained from Nanjing Animal Center. B6.SJL-CD45a(Ly5a) (CD45.1) mice were purchased from Peking University Health Science Center. *IL18***-/-**, *IL22-/-*, *caspase-1/11***-/-**, *NLRP3-/-*, and *NLRC4***-/-** in a B6 background were from Professor Meng at the University of Chinese Academy of Sciences (Shanghai) and Professor Shao at the National Institute of Biological Sciences (Beijing). All experimental litters were bred and maintained under SPF conditions at the Animal Center of Nankai University. The experiments were performed using age- and sex-matched mice. All procedures were conducted according to the Institutional Animal Care and Use Committee of the Model Animal Research Center. Animal experiments were approved by the Institute’s Animal Ethics Committee of Nankai University. All experimental variables such as husbandry, parental genotypes, and environmental influences were carefully controlled.

### Human Samples

For human samples, the samples from the male patients with UC, which were shown by pathologic analyses but not given clinical treatment, and from healthy individuals were collected by Tianjin Union Medical Center. Age information was not specifically collected for this study. This study was conducted with the approval of the Institutional Review Board at Nankai University. All participants provided written informed consent. The Mayo index (Mayo endoscopic score)^26^ is 0 points for normal or inactive lesions, 1 point for mild lesions (redness, reduced texture of blood vessels, and mildly brittle mucosa), 2 points for moderate lesions (significant erythema, disappeared texture of blood vessels, and brittle or eroded mucosa), and 3 points for severe lesions (spontaneous mucosal bleeding or ulceration).

### Analyses of Gut Microbiota

For colony analysis of the gut tract and gut tissues, the homogenized colon tissues from DSS-treated or unmolested mice or colon tissues of UC patients were harvested; then, we serially diluted the homogenates and plated them on bacterial media that support the growth of *E coli* such as Luria-Bertani. We then incubated the plates aerobically at 37°C for 24 hours, after which we counted the colonies, classified them based on colony appearance, and subjected them to 16S ribosomal DNA colony polymerase chain reaction and sequencing. The sequences were classified using Microbial Nucleotide BLAST (https://blast.ncbi.nlm.nih.gov/Blast.cgi).

### Bacterium Stains

We isolated *E coli* 0160:H7 from the DSS-mediated colitic tissues of mice.^27^^,^^28^ *KLPJ* was isolated from the colitis tissues of patients with IBD. These bacteria were grown in Luria-Bertani media shaken at 37ºC overnight and stored in 25% glycerol frozen stocks until used for experiments.

### Mouse Models

For the *KLPJ* infection model, mice were first treated with pan-antibiotics (ampicillin [A, 1 g/L; Sigma], vancomycin [V, 0.5 g/L; Sigma], neomycin sulfate [N, 1 g/L; Sigma], and metronidazole [M, 1 g/L; Sigma]) via drinking water for 1 week (sometimes longer than 1 week); then, they were orally administered 200 μL of 1 × 10^9^ bacteria (*E coli* or *KLPJ*) (once/wk). To confirm the elimination of bacteria, stools were collected from the antibiotic-treated and untreated mice and cultured in anaerobic and aerobic conditions. Mice were killed on the indicated days. Representative colon tissues were treated with flow cytometry and embedded in paraffin for H&E staining or embedded in OCT compound (Tissue-Tek; Sakura, Torrance, CA) for immunostaining.

DSS-induced colitis was induced according to our previously reported method,^52^ with modifications. Briefly, mice received 2.5% DSS (4 wt/vol; MP Biomedicals) for non–pan-antibiotics–treated mice, 2.0% for pan-antibiotics–treated mice, or at the indicated dose in their drinking water for 7 days; then, they were switched to regular drinking water. The amount of DSS water intake per animal was recorded, and no differences in intake between strains were observed. For the effects of *KLPJ* on DSS-induced colitis, the mice were first treated with pan-antibiotics and orally administered 200 μL of 1 × 10^9^ bacteria (*E coli* or *KLPJ*) (once/wk) for 1 week. DSS was given after 2 days.

For survival studies, the mice were followed up for 12 days from the start of DSS treatment. Mice were weighed every other day for determination of the percentage weight change. This was calculated as follows: percentage weight change = (weight at day X – day 0 / weight at day 0) × 100. The animals also were monitored clinically for rectal bleeding, diarrhea, and general signs of morbidity, including hunched posture and failure to groom. The DAI was the average of these scores, as follows: (combined score of stool consistency, bleeding, and weight loss)/3.^53^ Diarrhea was scored daily as follows: 0, normal; 2, loose stools; and 4, watery diarrhea. Blood in stool was scored as follows: 0, normal; 2, slight bleeding; and 4, gross bleeding. Weight loss was scored as follows: 0, none; 1, 1%–5%; 2, 5%–10%; 3, 10%–15%; and 4, >15%. For histologic evaluation, histology was scored as follows: epithelium: 0, normal morphology; 1, loss of goblet cells; 2, loss of goblet cells in large areas; 3, loss of crypts; and 4, loss of crypts in large areas; and infiltration: 0, no infiltrate; 1, infiltrate around the crypt basis; 2, infiltrate reaching the lamina muscularis mucosae; 3, extensive infiltration reaching the lamina muscularis mucosae and thickening of the mucosa with abundant edema; and 4, infiltration of the lamina submucosa. The total histologic score was given as epithelium + infiltration.

For the IL18 blocking model, mice were injected in the tail vein using IL18 blocking agents (IL18 Binding protein isoform d, 75 μg/kg); then, they were given 2% DSS in their drinking water for 7 days.

For the bone marrow cell transplant experiment, recipient mice were irradiated (800 cGy as a single dose) using a Shepherd Mark I Cesium Irradiator (J.L. Shepherd and Associates). Then, the bone marrow cells collected from WT or *IL18* KO mice were injected into the irradiated *IL18* KO or WT recipient mice (2 × 10^6^ cells per mouse) via the tail vein.

### Gut Organoid Culture

For the mouse small intestinal organoid culture, the previously reported methods were used in this study.^54^^,^^55^ Briefly, the crypts were incubated for 15 minutes at room temperature in gentle cell dissociation reagent; then, the crypts were passed through a 70-μm cell strainer and collected by centrifugation at 100 × g for 5 minutes. The crypts were embedded with Matrigel (Corning), plated, and incubated at 37°C for 10 minutes to allow the domes to polymerize. After polymerization of the Matrigel, IntestiCult (STEMCELL) organoid growth medium (mouse) was added and placed on the plate to incubate at 37°C and 5% carbon dioxide was used for incubation. The culture medium was fully exchanged 3 times per week. For the mice colonic organoid culture, the colonic tissues were separated and the crypts were incubated for 25 minutes at room temperature in gentle cell dissociation reagent; then, the detailed operations were the same as the intestinal organoid culture. For the human colonic organoid culture, human colonic tissues were obtained from patients at the Tianjin People’s Hospital. Fresh colonic tissues were washed with ice-cold phosphate-buffered saline (PBS) to remove adipose tissues and blood, and then incubated in a gentle cell dissociation reagent for 25 minutes at room temperature. The crypts were enriched by centrifugation and embedded with Matrigel and plated. After full Matrigel polymerization at 37°C, 750 μL complete human IntestiCult organoid growth medium supplemented with 10 μmol/L final concentration Y-27632 was added for primary culture. Then, we placed the lid on the culture plate and incubated it at 37°C and 5% CO_2_. Every 2 days, a full medium change was performed with complete human IntestiCult organoid growth medium (Y-27632 was not required).

For the entry of bacteria and organoid immunofluorescence staining, disaggregated organoids from Matrigel with dispase (1 mg/mL) were resuspended in 200 μL Matrigel, plated into glass-bottom microinjection dishes (INTRAcel; Willco Wells), and covered with gut organoid base growth medium. The Eppendorf TransferMan NK2-FemtoJet express system (Eppendorf) was used to inject bacteria (10^7^/mL/mL) into organoids. All injections were performed in a chamber at 37°C and 5% CO_2_. After injection, organoids were incubated for 3 hours at 37°C and 5% CO_2_ and then fixed for microscopy. For immunofluorescence staining, bacteria-injected organoids were collected and gently spun at 100 × *g* for 3 minutes. The organoids were fixed with 1 mL 4% paraformaldehyde for at least 1 hour at room temperature or 4°C overnight. The organoids were washed 3 times with 1× PBS and gently spun at 100 × *g* for 3 minutes and allowed to settle to the bottom of the tube by gravity after each wash. The organoids were blocked with 1× blocking buffer (1 × PBS + 5% goat serum + 0.3% Triton X-100 [Life Technologies]) for 1 hour at room temperature, and the organoids were incubated with primary antibodies in antibody dilution buffer (1 × PBS + 1% bovine serum albumin + 0.3% Triton X-100) overnight at 4°C. After washing the organoids 3 times with 1× PBS for 10 minutes each wash, the organoids were incubated with secondary antibodies with 1× secondary antibody buffer (1 × PBS + 0.3% Triton X-100) for 2 hours at room temperature in the dark. The nuclei were stained with 4′,6-diamidino-2-phenylindole (DAPI). Finally, the organoids were carefully pipetted in a barrier pipette tip onto raised chamber slides and observed using a confocal microscope.

For stimulation on gut organoids, gut organoids were removed from Matrigel with dispase (1 mg/mL) for 30 minutes at 37°C, and then suspensions of gut organoids were prepared following dissociation of the cells by pipetting with a P1000 pipet and passing the cells through a 40-μm cell strainer. The cells were pelleted for 5 minutes at 400 × *g*, suspended in 100 μL complete medium, and then were stimulated with *KLPJ*, *E coli* (1 × 10^7^ CFU/mL^−1^), or LPS (final concentration, 1 mg/mL). Stimulated organoids were incubated for 3 hour at 37°C and 5% CO_2._ Cell lyses then were prepared and analyzed by immunoblotting for proforms and mature forms of caspase-1 and IL18.

### Ex Vivo Stimulation

For ex vivo colon stimulation, colons from healthy mice were harvested, washed, and incubated with or without 1–2 × 10^8^ *E coli* or *KLPJ* in Dulbecco’s modified Eagle medium (DMEM) with adenosine triphosphate (2 mmol/L) for 1 hour. For analyses of caspase-1, caspase-11, and IL18, the colon epithelial cells were separated from the colon tissues using 0.1% EDTA, followed by 1 minute of shaking by hand 3 times, a 15-minute incubation at 4ºC, and passage through 70-μm filters (BD Falcon). A fraction containing intact and isolated crypts was collected by centrifuge at 75 × *g* for 5 minutes at 4ºC and washed with PBS. The lamina propria was separated from the crypts to enrich for mononuclear and intestinal epithelial cells, respectively. The cells were lysed with cell lysis buffer (Cell Signaling Technology), which was supplemented with a protease inhibitor cocktail (Calbiochem). Protein extracts were analyzed by immunoblotting for pro- and mature forms of caspase-1, caspase-11, and IL18. The supernatants were collected, and the expression of caspase-1, caspase-11, and IL18 was analyzed using immunoblotting and/or enzyme-linked immunosorbent assay (ELISA).

### Cell Isolation and Flow Cytometry

For the staining of lamina propria immune cells, the mice were killed using carbon dioxide; then, the colons were removed and transferred immediately onto laboratory tissue paper soaked liberally in PBS (no calcium or magnesium, at room temperature). The colons were opened longitudinally and washed vigorously by agitation in a PBS-filled Petri dish to remove the colon content. Fine forceps were used to gently squeeze out any remaining mucus; then, the colon was cut into 1 mm^3^ pieces. The samples were poured onto Nitex mesh and washed with nonsupplemented Hank’s balanced salt solution. After the final wash, the colon pieces were transferred into 15 mL digestion buffer (DMEM, 5% fetal bovine serum, 1 mg/mL collagenase IV [Sigma-Aldrich], and DNase I [Sigma-Aldrich]) for 40 minutes. After the 15-minute digestion period, the enzyme action was stopped rapidly by adding 35 mL ice-cold DMEM. The digested tissues then were filtered through a 40-mm filter. The samples were spun at 400 × *g* for 10 minutes at 4°C to pellet the isolated cells. The cells were resuspended in 10 mL of the 40% fraction of a 40:80 Percoll gradient and overlaid on 5 mL of the 80% fraction in a 15-mL Falcon tube. Percoll gradient separation was performed by centrifugation for 20 minutes at 1800 rpm at room temperature. Lamina propria immune cells were collected at the interphase of the Percoll gradient, washed, and resuspended in medium for staining.

For surface staining of different immune cell populations, the cells were washed with staining buffer containing PBS, 2% fetal bovine serum (FBS), 1 mmol/L EDTA, and 0.09% NaN3. After eliminating doubles by Flow cytometry Standard-W and SideScatter-W, and dead cells by 7-aminoactinomycin D, CD45^+^ cells were gated. Surface markers on these cells were analyzed further with fluorescein isothiocyanate, Phycoerythrin (PE), Allophycocyanin (APC), perCP/Cyamine 5.5 or other color-labeled anti-CD4, CD11b, Ly6G, MHCII, Ly6C, and/or NKp46 antibodies.^56^ After staining, the cells were analyzed by FACScan flow cytometer (BD Biosciences).

For surface and intracellular staining, the cells were cultured and stimulated for 6 hours with 50 ng/mL phorbol 12-myristate 13-acetate (Sigma) and 1 μg/mL ionomycin (Sigma) in the presence of GolgiStop (10 ng/mL; BD Biosciences). After incubation for 6 hours, the cells were washed in PBS, stained for surface maker, and then fixed in Cytofix/Cytoperm, permeabilized with Perm/Wash buffer (BD Biosciences), and intracellularly stained with fluorescein isothiocyanate, PE, APC, PerCP/Cy5.5, or other color-conjugated antibodies. After staining, the cells were analyzed by FACScan flow cytometer (BD Biosciences).

For splenocyte staining, the splenocytes were obtained and collected from the chimera mice, rinsed twice with ice-cold PBS, and then incubated with APC-CD45.1 and PE-CD45.2 antibodies for 30 minutes in PBS with 1% FBS. After washing twice, cells were resuspended in PBS and analyzed using FACScan flow cytometer (BD Biosciences).

### H&E Staining, Immunostaining, and Fluorescence In Situ Hybridization

For H&E staining, the previously reported methods were used in this experiment.^52^^,^^56^ Briefly, the entire colon was excised to measure the length of the colon; then, it was fixed in 4% (w/v) paraformaldehyde-buffered saline and embedded in paraffin, and 5-μm sections were cut and stained with H&E.

For immunostaining, the samples were prepared from frozen tissue and fixed in acetone (−20°C) for 10 minutes. After rehydration in PBS for 5 minutes and further washing in PBS, the samples were blocked with 1% (w/v) bovine serum albumin and 0.2% (w/v) milk powder in PBS. After TRIS-EDTA treatment, the primary antibody was added in PBS with 1% goat sera and incubated overnight at 4°C. After PBS washing (3 times, 5 minutes each), the tissue was detected with a 3,3′-diaminobenzidine tetra hydrochloride kit or fluorescence-labeled second antibody. The nuclei were stained by DAPI.

For fluorescent in situ hybridization, mucus immune-staining was paired with fluorescent in situ hybridization to analyze bacteria localization at the surface of the intestinal mucosa according to the previously reported method.^57^^,^^58^ In brief, colonic tissues were placed in methanol and Carnoy’s fixative solution (60% methanol, 30% chloroform, and 10% glacial acetic acid), for a minimum of 3 hours at room temperature. The tissues then were washed in methanol, ethanol, ethanol/xylene (1:1), and xylene, followed by embedding in paraffin with a vertical orientation. Then, 5-μm sections were cut and dewaxed by preheating at 60ºC for 10 minutes, followed by bathing in xylene at 60ºC for 10 minutes, xylene at room temperature for 10 minutes, and 99.5% ethanol for 10 minutes. The hybridization step was performed at 50ºC overnight with a probe diluted to a final concentration of 0.01 μg/mL in hybridization buffer (20 mmol/L Tris-HCL, pH 7.4, 0.9 mol/L NaCL, 0.1% sodium dodecyl sulfate, 20% formamide). After washing for 10 minutes in wash buffer (20 mmol/L Tris-HCL, pH 7.4, 0.9 mol/L NaCL) and 10 minutes in PBS, a block solution (5% FBS in PBS) was added for 30 minutes at 50ºC. The nuclei were stained using DAPI. Observations were performed with a Zeiss LSM 700 confocal microscope with Zen 2011 software (version 7.1).

### Immunoprecipitation and Immunoblot

Immunoprecipitation and immunoblotting were performed according to previously reported methods.^52^^,^^56^ The cells were lysed with cell lysis buffer (Cell Signaling Technology), which was supplemented with a protease inhibitor cocktail (Calbiochem). The protein concentrations of the extracts were measured using a bicinchoninic acid assay (Pierce).

Immunoprecipitation was performed according to our previously published method.^59^ The gut epithelial cells were lysed in immunoprecipitation lysis buffer (Pierce, Rockford, IL) containing 10% phenylmethylsulfonyl fluoride. Protein A/G magnetic beads (Pierce) were first added into the cell lysates for preclearing. The supernatant was collected after centrifuging at 12,000 rpm and then immunoprecipitated overnight at 4°C with the antibodies or control antibodies. Protein A/G magnetic beads were added into the cell lysates and incubated for an additional 3 hours. After being washed 5 times, the lysates were denatured and resolved by sodium dodecyl sulfate–polyacrylamide gel electrophoresis.

For immunoblotting, hybridizations with primary antibodies were conducted for 1 hour at room temperature in blocking buffer. The protein–antibody complexes were detected using peroxidase-conjugated secondary antibodies (Proteintech) and enhanced chemiluminescence (Millipore).

### ELISA

To determine IL18 levels in the supernatants of the gut tissues or organoid, ELISAs were performed according to the manufacturer’s protocol. To determine tissue levels of cytokines, the frozen tissues were homogenized in lysis buffer (PBS, 1% Triton X-100, and protease inhibitor) using a Power Lyser 24 bench top bead-based homogenizer (Mobio). The lysates were centrifuged. The supernatants were used for ELISA.

### Statistical Analyses

The 2-sided Student *t* test and 1-way analysis of variance Bonferroni multiple comparison test were used to determine significance. The statistical significance of the survival curves was estimated using the Kaplan–Meier method, and the curves were compared using the generalized Wilcoxon test. Histologic scores were analyzed by the Mann–Whitney *U* test. A 95% CI was considered significant and was defined as *P* < .05. ∗*P* < .05, ∗∗*P* < .01, ∗∗∗*P* < .001.

## CRediT Authorship Contributions

Rongcun Yang, Doctor (Conceptualization: Lead; Funding acquisition: Lead; Project administration: Lead; Supervision: Lead; Writing – original draft: Lead)

Qianjin Zhang (Data curation: Lead; Methodology: Lead)

Xiaomin Su, Doctor (Data curation: Lead; Funding acquisition: Supporting; Methodology: Lead)

Chunze Zhang (Data curation: Lead; Methodology: Lead)

Wei Chen (Investigation: Supporting)

Xiaorong Yang (Investigation: Supporting)

Dan Liu (Investigation: Supporting)

Yuan Zhang (Investigation: Supporting)

Ya Wang (Investigation: Supporting)
